# The Ecological Genome Project and the Promises of Ecogenomics for Society: Realising a Shared Vision as One Health

**DOI:** 10.1111/bioe.70020

**Published:** 2025-07-24

**Authors:** Benjamin Capps, Ruth Chadwick, Yann Joly, Claire Lajaunie, Iva Hauptmannova, Susannah Mackenzie, John. J. Mulvihill, Elizabeth Mumford, Sonja A. Rasmussen, Kunal Sanghavi, Donrich W. Thaldar, James Yeates, Maud C. Quinzin, Zohar Lederman

**Affiliations:** ^1^ Department of Bioethics Dalhousie University Halifax Nova Scotia Canada; ^2^ School of Social Sciences Cardiff University Cardiff Wales UK; ^3^ Centre of Genomics and Policy, Institute of Genomics Medicine McGill University Montreal Quebec Canada; ^4^ Inserm, Aix Marseille University, IRD, LPED Marseille France; ^5^ Humanimal Trust Eashing Surrey UK; ^6^ Schulich School of Law Dalhousie University Halifax Nova Scotia Canada; ^7^ University of Oklahoma Health Sciences Oklahoma City Oklahoma USA; ^8^ National Human Genome Research Institute Bethesda Maryland USA; ^9^ School of Veterinary Medicine and Institute for Sustainability University of Surrey Guildford UK; ^10^ Department of Genetic Medicine Johns Hopkins University School of Medicine Baltimore Maryland USA; ^11^ The Jackson Laboratory for Genomic Medicine Farmington Connecticut USA; ^12^ School of Law University of KwaZulu‐Natal Durban South Africa; ^13^ World Federation for Animals Boston Massachusetts USA; ^14^ World Maritime University Malmö Sweden; ^15^ Department of Emergency Medicine University of Hong Kong Hong Kong

**Keywords:** conservation, ecogenomics, ecology, environmental, genome, one health

## Abstract

This paper develops a vision for *The Ecological Genome Project*: an aspirational, global endeavour to connect human genomic sciences with the ethos of ecological sciences. The Project's goal is to strengthen interdisciplinary networks that relate to diverse initiatives using genomic technologies, with respect to shared ethical frameworks and governance structures. To this end, this paper proposes a practical definition of ecogenomics to align various methodologies and values in a single environmental field using principles used to safeguard all forms of life in their habitats. We achieve this by using a One Health approach as a pretext for disparate disciplines to collaborate and also a lens to view the Ethical, Legal and Social Implications (ELSI) inherent in ecological systems.

## 1 Introduction

In 2023, the Human Genome Organisation's (HUGO) Committee on Ethics, Law and Society (CELS) suggested that an interdisciplinary One Health (OH) approach should be adopted in genomic sciences [1]. OH is defined as ‘an integrated, unifying approach that aims to sustainably balance and optimize the health of people, animals and ecosystems’ [2]. As such, it can provide a common language and knowledge framework that underpins environmental research [3]. In this paper, we revisit the concept of *ecogenomics* as part of a response to an evolving ‘nature crisis’, which has now been recognised by more than two hundred health journals as a systemic ‘global health emergency’ [4]. The crisis includes unprecedented anthropogenic biodiversity loss and environmental deterioration; it is an inherently OH problem to address, through collaborations between disparate fields, creating disciplinary networks, and forming cross‐cultural dialogues. Genomics is critical to monitoring and restoring healthy ecosystems [5], and to this end, we argue for integrating the worlds of human and ecological sciences. We coin the *Ecological Genome Project* as an aspirational initiative to form the basis for these collaborations. This paper considers how such collaborations might shape theory, practice and governance in genomics.

In 2024, the authors convened at the Brocher Foundation, on the shores of Lake Léman near Geneva, Switzerland, to explore the ‘promises of ecogenomics for society.’ The workshop was an experiment in OH: to collaborate with experts not normally associated with HUGO and to sympathetically engage with environmental ideas that are nontraditional to the field of genomics. Our group included bioethicists, clinicians, conservationists, genetic counsellors, lawyers, public health epidemiologists, veterinarians and social scientists. The context of this meeting shaped the focus of the following analysis. In the first section, we use ‘the environmental genome’ to bring into focus the Ethical, Legal and Social Implications (ELSI) inherent in ecological systems; this sea change has practical implications for HUGO because of the commonality of anthropogenic environmental impacts. Next, we use OH to frame *eco*logical sciences and human *genomics* as a single field engaged in environmentally relevant phenomena. In part three, three areas are illustrated as future enquiries necessary to reconcile effective collaboration: exploring theories of environmentalism, applying bioethics in genomics, and developing governance. Each area provides a glimpse of the possibilities resulting from enhanced contact and coherence between human genomic and ecological sciences. Finally, we explore how OH and ecogenomics inform two existing discourses in public health and bioethics.

## 2 An (Underexplored) Environmental View of the Genome

John Sulston, one of the architects of the Human Genome Project (HGP), wrote that it would be ‘a watershed in the history’ [6] of the data‐driven Genomic Era, premised on the belief that ‘Somewhere in the genome will be the answer to what makes us different from all the other species—what makes us human’ [6, p. 259]. This dogma defined an *ecogenetic* approach to advance the field of public health genetics [7]. The first major initiative to include the environment in genomics was the US National Institute of Environmental Health Sciences’ *Environmental Genome Project*. Launched in 1997, its goal was to systematically sequence *human* genetic variants to understand environmental exposures at the population level [8]. Here, ‘ecology’ was used to refer to environmental triggers for genetic conditions and lifetime exposures to exogenous agents. The methods focussed mostly on metagenomics (sequencing the organisms in the environment that affect human health) and the environment's impact on mechanisms involved in gene expression, such as epigenetics. Today, this study is fuelled by biobanks that include multi‐species genomic sequences, such as the ongoing *Earth BioGenome Project*: a ‘moonshot for biology’ [9] that will eventually contain sequences of all ~1.8 million eukaryotes reference genomes. As well as a logical step in developing comprehensive mechanistic models of gene–environmental interaction, the Project's planners justify it as a ‘global imperative for human survival and prosperity’ [9, p. 4326] and its benefits to humanity [10]. However, despite knowing the environmental context of the interplay between human and nonhuman genomic elements, there is a perception that ethics and governance still converge on public health – ecological scientists (merely) raise some ‘moral hazard concerns [that] are unlikely to be borne out’ [11]. This approach to environmental public health [12] has a familiar ring to the ethical principles formulated through ELSI initiatives [8] and now underpins the environmental justice movement's approach to community and population health [13]: that is, environmental exposures can be managed by minimising significant risks, and these risks are constructed in ways that differentiate among population strata [14]. That view, perhaps, underplays the challenges of practically combining human genomics with the aims and methods of ecological sciences, and the potential for conflicts between conservation goals or protecting environments, with public health. Sensibly, all ecogenomic projects identifying, extracting and sequencing genomes in nature ought to incorporate environmental views, but too often, public health becomes a justification in and of itself, without recognising the potential value tensions arising [5].

### 2.1 Ecogenomics

A point to stress is that approaches grounded in public health may overlook the wider impact of human actions on environments. The nature crisis underlies a concordant anthropocentricity, and that leads to interventions prioritising human health sometimes at a cost to a particular species (e.g., culling) or the system as a whole (e.g., widespread insecticide spraying) [15]. Adapting health systems to consider contexts of ecology is increasingly appealing: the Intergovernmental Science‐Policy Platform on Biodiversity and Ecosystem Services and the Intergovernmental Panel on Climate Change have suggested the need for ‘shifting values related to the human‐nature relationship’ [16]. Such connections can be viewed through the structures, functions and evolutions of ‘the environmental genome,’ to include the significance of ‘healthy’ eukaryotes, prokaryotes and environments, particularly with respect to the complexity of multispecies ecosystems and abiotic environments.

Viewed through this lens, healthier ecosystems are less likely to play a part in illness caused through stress responses and mutagenesis [17] and positively support the well‐being of communities within them. Genomic technologies can be used to discover populations and species, (re)introduce ones to re‐establish ecological processes (e.g., herbivore ‘engineers’ to support the carbon cycle), or select organisms (e.g., microbes and fungi) to decontaminate and revive rivers and soils. Technologies such as gene editing may be used to develop biocontrols for vectors and invasive species or to rescue populations to prevent extinctions [18]. Such uses can be controversial, causing conflict at the intergroup level, and may have unwanted, unexpected and uncontrollable effects on ecosystems. Genomics also raises implications of animal welfare: engineering environments and gene editing creatures carry risk, and are often technological responses to industries, for example, of intensive farming, that must be examined through the lens of unethical exploitation.

‘Ecogenomics,’ Robert W. Chapman proposed in 2001, is not focused upon the molecules and genomic processes—as ‘they are component parts, but hardly the whole picture’ [19]— but rather the ecological–social ecosystems that underlie intraspecific diversity and adaptive genetic variation [17, p. 381]: ‘At the core of EcoGenomics is the belief that the bewildering array of interactions between species and their environments can ultimately be understood in the same terms as the complex interactions of genes and proteins at the cellular level’ [19, pp. 549–550]. It has more in common with studies of ecosystems *and* the relationships between organisms in those systems [20]. As more species are sequenced and stored, there are opportunities that characterise an approach that aligns environmental and human genomic resources. But any successful genomic–environmental project will require synergies of values across the scientific–public interest–community nexus that are reflective of Sulston's dogma: perhaps rather than genetically unique, somewhere in the genome will be the answer to what makes us *similar* to other species—what makes us part of nature.

What might ‘the environmental genome’ look like? It is not one thing, *a* reference genome or a single project. DNA can be considered as a link between all life on Earth and the environment, and ‘the environmental genome’ is the metaphorical connection between health and the environment described in the genomes sequenced. Therefore, ecogenomics is a field to study the connections, scales and relationships, across species and shared spaces, which need to be ethically and legally understood, but as such, are so far underexplored in ELSI initiatives.

## 3 A One Health Approach

The generative origin of OH in conservation suggests that animals—human beings and nonhuman animals—are conceptually *indistinct* from the environment and from the plants, microbes and fungi found there: these are all parts of the ecosystem. The novelty of the approach was to address health through the connections among all forms of life as well as the abiotic environment; it implied that it is ‘increasingly difficult to continue to believe [the dogma] that nature is a completely separate domain from social life’ [21]. OH is an opportunity to broaden discussions of human genomics to include the bioethical and governance issues of *ecological sciences* [15]. It is easiest to define these disciplines as neither focused entirely on the health of human (beings) nor exclusionary of (non‐)human interests, with respect to ‘the environment,’ ‘nature’ and ‘ecologies’, of the places where beings are connected. We use these terms interchangeably, realising that there are methodological nuances; and, in this paper, we focus on veterinary medicine, conservation and ecology, recognising that ecological sciences also include all biotic (including plant sciences), abiotic and environmental sciences.

Each discipline will contextualise these connections differently. Conservationists generally place *all* animals and plants in the context of the environment, where health is defined by connections across all forms of life co‐existing in a given ecosystem. Ecologists see humans as one of the many biotic beings that are constituent parts of the ecosystems studied; their focus is on ecological problems across time spans and defined geographical areas. Veterinarians practicing ‘One Medicine’ see nonhuman animals as deserving of fair access to healthcare, premised on the long‐established approach to human and animal health as *one* field [15]. This movement to orient comparative and translational medicine towards the social value of healthy domestic and wild animals underlies the contemporary OH approach, too, as a practical goal of ‘safeguarding animal health and welfare [which is] also necessary to secure the well‐being of current and future generations of humans’ [22]. The Planetary Health movement, which often borrows methodologies from OH, seeks to reform medicine in response to years of environmentally unsustainable practices [23]. In these respects, then, OH cannot practically form the basis for future collaborations without consideration of complex bioethical dimensions. The OH approach suggests that there are no general or model solutions, but that there must be a response to unjustified hierarchies of knowledge so that it can be mutually shared to benefit humans, animals and environments.

### 3.1 Defining Ecological ELSI

From the view of ecogenomics, the nature crisis can be approached through governable social–ecological systems in which the accelerating loss of biodiversity on Earth is driven by human activities like unsustainable farming and deforestation, and consequential effects such as pollution and climate change: these systems are normative [24]. Within them, different branches of knowledge also flourish: each discipline plies their trade, based on specific languages, methods and measurements. Often, these disciplines have different priorities, and, in some circumstances, OH becomes not a solution in and of itself, but a tool for practical engagement between disparate theories and justifications. Interdisciplinarity can achieve some superficial commonalities with respect to the implications of the *genetic microcosm*: the same mechanisms of gene expression, inheritance and evolution that underlie the interactions between genomes and environment. However, human genomics and ecological sciences (e.g., conservation genetics) may sometimes be perceived to be isolated by different practical goals [25]; ‘broad and useful diversity’ in fact needs ‘more fruitful contact and coherence’ between them [26]. But that makes matters more contentious, especially where humanistic sciences must engage with a multitude of positive ‘human–nature relationships’ that include indigenous and community‐based knowledge [16, pp. 164–165], as well as the philosophies underlying ethical governance. The OH approach also draws attention to the concerns with respect to the influences of politics and economics; both are dominant and pervasive factors shaping ‘unjust’ environments. So, according to some models, interdisciplinarity is only practicable if seeking a common ethical purpose [26].

An OH approach potentially leads to conflict in the practice of public health separately from ecological sciences, even though there are commonalities (e.g., balancing the understanding that global warming raises sea levels that displace communities, but also kills corals). We therefore propose that the Ecological Genome Project grounds ‘a need for an interdisciplinary dialog and stronger links between conservation, health and the social sciences’, which are likely achievable by ‘sensitizing health practitioners [, including human health researchers], to issues of local ecology’ [27]. Moreover, the Project will engage with system sciences and ethics to identify methodologies that equitably balance trade‐offs across communities [28]. To achieve this, local communities will have to be meaningfully involved, and these may embody conservation, sustainability and responsibility towards benefitting from ecoservices, through respect of their nonhuman inhabitants [29]. Our vision is of a multidimensional project that is adaptable and cooperative, and is a collective response to environmental problems. The common purpose is responding to anthropogenic climate changes and biodiversity loss that are contributing to the nature crisis. We encourage a constant dialectic of the impacts of the environment on the genomes and behaviours of and across species in the places they are found. By studying these interactions at all levels, from molecules to phenotypes and genotypes, and developing an understanding of their functions in ecosystems, we can respond to the shaping of environments by human beings and vice versa. But that also must factor in *normative* solutions based on the local and global interaction and movement of beings and transmission of ideas, the trans‐political nature of boundaries, and the scope for practical disciplinarity between the plural communities.

## 4 Three Enquiries

### 4.1 Environmentalism

How can we articulate a joint vision of the Ecological Genome Project? Ecological sciences each have a long bioethical precedent grounded in *environmentalism*: that concept can take on a specific (dictionary) meaning that relates to protecting the environment from human activities [15]. The concept in essence describes the experiential influence of the environment upon a person, including its effects on their genome. Theories of environmentalism also imply an ‘‐ism’ property, concerned with the circumstances, place and context—‘[…] a set of cultural and political responses to a crisis in humans’ relationships with their surroundings’ [30]. But if the coinage of ‘the environment’ was meant to question a binary lens to view culture and nature (which, with respect to the topic at hand, has a familiar ring to the genetics lens of nature/nurture), then *all* organisms have biological responses and experiences that relate to their surroundings. Fundamentally, then, ecogenomics is an opportunity to trace the roots of ethical environmentalism, while also being part of the contemporary environmental justice movement.

Understanding how existing practices and policies affect the environment, in terms of both its effects on human interests and how to orient spaces shared with all other species, will require an approach capable of understanding and assimilating diverse social science methods and theories in environmental ethics [31]. As such, ecogenomics will be a normative project that challenges existing views that limit the imagination in understanding ecosystems, where the traditional frame of *bio*ethics specifies the terms, conditions, opportunities and costs of living with nonhuman organisms and respecting nature. As such, we should make space in the ‘promises of ecogenomics for society’ [32] to recognise that public health and population genomics have implications for the health of nonhuman species as well. That means, too, we should expect conflicts, as questions of the public interest and animal rights can divide communities just as much as they unite them.

### 4.2 (Clinical) Genomics

What bearing does ecology have on understanding medical research and clinical applications of genomics? The field of ecogenetics established that abiotic as well as biological and social environmental factors have an impact on human development, and assessment of lifestyle and environmental exposures now informs the overall clinical evaluation for a patient [33]. This environmental narrative suggests that these impacts, which often do not affect all populations equally [14], will be ever more prevalent unless environmental stresses are reversed and ecosystems are restored [34].

Ecogenomics expands the scope for direct and preventive interventions in a landscape where ‘orthologous gene sets [from different species] will illuminate the single tree of life on our planet, the study of which can potentially unify researchers studying different species around the common core of all biology’ [35]. The long recognition of gene–environmental interactions that likely underlie some human cancers [36], and the ensuing population health approach to environmental exposures [37], is part of a ‘One Medicine’ approach integrating veterinary medicine in community health.^1^ Here, the ‘lifetime’ exposome^2^ and predictive approaches to such diseases^3^ underlie the environmental factors that cross between species and a potentially fruitful field for training (medical and veterinary) and coordination (in environmental public health). *Environmental DNA* (eDNA)—genetic material found in waters and soils—also includes human DNA traces [42]. But the focus to date has been on sequencing environmental samples to find out what is (or was) ‘out there’ normally as pathogens and vectors, and capturing genetic resources; ecogenomics puts such sequencing to work in restoring and conserving multi‐genomic‐connected environments. Similarly, epigenetics—extra‐DNA modifications controlling gene expression—is increasingly assessed in clinical research of various disorders relatable to population health, including as a result of migration and displacement from anthropogenic climate change and natural disasters. So, adapting to new environments and reconstruction of destroyed ones—from seas rising to places decimated by war—must consider the social–genomic relations formed in new environments. Many of these climate and displacement indicators are traceable in the genomes of both displaced animals and humans, and underlie the adaptability and the evolution of biotic pathogens [43].

### 4.3 Genomic Governance

In the moment of recognising the nature crisis, it appears opportune for research communities to redefine how they think about the interstice of international law and the environment. A paradigm that merges the regulatory frames of clinical genomics and theory must adapt to the ethics, histories and sociologies of ecological sciences. Interspecies biobanks may promote interdisciplinarity, which could lead to cross‐diagnosis (exposures found across species) and justify collective (not just public) health interventions. Veterinarians, conservationists and ecologists drawing on resources once particular to clinical genomics may require such institutions to adopt new ELSI principles to justify diverse methodologies: nonclinical or interspecies population health research and legal regimes will need to adapt. eDNA initiatives sequencing pathogenic genomes raise access risks and dual use. And, genomic technologies may be used to fundamentally change environments—either wisely or in error [44].

An OH approach might encourage interprofessional discussions of the ethical trade‐offs between clinical health and public health (e.g., antibiotic resistance is also an environmental matter) towards an understanding of sustainable ecological, as well public‐health systems [45]. While acknowledging that ‘reversing the loss of biological diversity, for the benefit of all living beings, is a common concern of humankind’ [46], there are few actionable and interoperable international norms or proven effective governance methods that are *interspecific*; instead, they separate human rights from animal welfare and describe the experiences of environmental disasters as anthropocentric. Indeed, many obstacles currently impede the development of an equitable framework for OH: the governance aspects of balancing the interests of all elements—human, animal, environmental—is a moral question as well as a legal one, and the law must not shy away from such questions [47]. There are opportunities, for instance, to develop genomic governance in ways that bridge the divides between clinical oversight, animal welfare and environmentalism, which will need reconsideration of the meaning of ‘human’ and ‘human rights’ towards more inclusive concepts [48]. Great care should be put into framing these nascent regimes in a manner that will promote collaboration and interoperability between disciplines (e.g., sharing of genomic data and ‘dual use’ conditions for data access).

Moreover, legal reasoning needs to account for the human footprint on the land and anthropogenic cascades that unregulated activities cause—at the International Criminal Court, there are efforts to transform the world's response to environmental destruction by making *ecocide* a punishable criminal offence [49]. The law might now extend protections to interspecies concepts of sustainable and dynamic environments, recognising that every species responds to others: this requires a whole‐of‐ecosystem legal approach, of which humans are just one part.

## 5 Epilogue: A Bioethical Clearing House?

The HGP‐centred ELSI research; the simplicity of a single, composite reference genome has been replaced by a complex vision of the pangenome and the genetic variation within populations [50]. It has long been observed that ‘the environment’ interacted with the heritability of complex traits in communities [51], which is today an eco‐social concept of the exposome. The mid‐Twentieth Century ‘environmental crisis’ recognised the effects of pollution on human health as part of a social–ethical critical lens for environmental justice. However, the concurrent disappearance in biodiversity was indicative that ‘time is running out’ for sustainable living on planet Earth [17, p. 380]. Ecogenomics became a vision to ‘…translate this [biodiversity loss] into an understanding of the responses and interactions of organisms to the environment and to one another’ [19, pp. 549–550]. In this paper, we have justified ecogenomics as a moral environmentalism and sown the seeds for a practical Ecological Genome Project. To realise this vision, bioethics must be re‐centred in two areas.

One, the Ecological Genome Project recognises the similarities (evolutionary) and variance of the ecogenome (a ‘natural pangenome’) to benefit both human beings and animals. Van Rensselaer Potter's ‘Global Bioethics,’ inspired by Leopold's *Land Ethic*, was ‘a long‐term view that is concerned with what we must do to preserve the ecosystem in a form that is compatible with the continued existence of the human species’ [52]. In bioethics, the tension between cultural and natural was present from the start [53], but recognition of its environmental heritage became obscured by a *medical* ethics lens that had a sense in which the ‘…*summum bonum* of preserving trees has no place in an ethic of social justice’ [54]. The formative ‘*bio*‐*ethical*’ lens ought to be once again focused on humanity, animals and environments, necessitating an OH inclusive of ethical frames in ecological and conservation sciences. Massive disruptive events, such as pandemics, climate change and natural disasters, are also experienced by animals and change ecosystems, and genomics plays a role in community responses, recovery and adaptation. In other words, biodiversity should be valued and preserved not only for its benefits to humankind.

Two, public health responses can lead to tragic choices (e.g., culling) [55] and socio‐ecological controversies (e.g., using gene drive to genetically modify or extirpate a species, such as mosquitoes) [44] if analysed in OH terms. In these cases, voices of ecologists, conservationists, zoologists and others ought to amplify the natural implications as phenomena that frequently relate to ethics, law and economics. The catastrophe of highly pathogenic avian influenza (HPAI), already panzooic in birds and nonhuman mammals, will have an impact on every pandemic policy and recommendation in far more complex ways if viewed with an OH lens [15]. These challenges cannot be circumvented only by inclusion of more disciplines: experts in ‘zoological, veterinary, microbiological, animal health, ecology and other cognate disciplines’ were confident that intensifying livestock production could reduce certain risks of zoonotic emergence [56], and yet, their certainty reflected an absence of perspective from ‘economics, geography, anthropology, political ecology, ethics or other social science disciplines’ [57]. But neither study alluded to the ecogenomic lens that we have developed here. Our critique therefore extends the concepts of molecular biology even beyond typical socioeconomic constructs^4^ and likely develops in ways that challenge the rank and file of ‘practitioners rather than philosophers [*qua* bioethics, who] may [feel they are]…better placed to understand the political and strategic climate in which public health decision making operates’ [59]. There are blind spots in such apparent public authority: the imperative to sequence genomes carries with it risks, and the resources may not solve the animal health and welfare issues that create worse circumstances for all. Universalising access to sequencing data of multiple species, for example, means that someone could utilise the sequences to ignite an unnatural pandemic.^5^ We must admit, then, that matters can also become truly unmanageable in a quest to hear from *everyone*, and in circumstances where *every and all data are open*, it risks bioethical dogmatism [62].

Thus, we envision a clearing house seeking the benefits of and for nature: an intermediary for the exchange of healthcare, ecological, conservation and veterinarian data and ideas [34]. We do not say here what that should be. But bioethics must get its own house in order, too: it needs to take seriously OH as a theory of environmentalism and environmental justice, uniting divergent interests ‘…often competing for attention and funding for the programmatic priorities… that reflect very different institutional logics, power structures and agendas’ [63]. The ongoing genomic projects have become the lens to justify sequencing and collection of very large data sets of many species in which ‘…great advances are made by teams of specialists with a desire to speak a common language and united in seeking a common goal’ [19, p. 551]. If these simply circle back to the ethics of the HGP, then, our recentring will fail our vision of *consilience*: an approach to seek convergent ethical principles across different disciplines of science and humanities and recognising their separate values, methods and epistemologies [19]. Stephen J. Gould used the metaphor of the ‘hedgehog’ to understand genomics as a unifying idea, warning us that such an animal reacts to threats every time in the same way, by rolling up into a prickly ball. He cautions us, too, about the slippery relativism of the ‘fox’: without *reason* for finding what is ‘in common,’ we are unlikely to achieve social coherence [26]. Rather than a field that tips the balance further towards public health activities, ecogenomics is both an academic *and* a practical endeavour developed with the overarching goal of securing universal goods between humans and animals in the environments that they share [64]. We have suggested that OH provides one such reason.

## 6 Conclusion

A 2023 CELS paper [1] closed with the words of John Sulston (and co‐editor, Georgina Ferry), who wrote that HUGO was historically ‘interested primarily in medical genetics rather than wider biological importance of genomes’ [6]. The present authors believe that HUGO's role could evolve as well. The HGP has already become more than the sequencing of the human genome and its clinical context; here, we presented an opportunity to explore ELSI with respect to ecosystems, environments and communities. Our goal has been to advocate for widening the study of reciprocal interactions between genomic theory and empirical observations from the field, laboratory and clinic. If, as the authors have argued here, OH is a view of environmentalism, then the approach also links the ethical, legal and social determinants of health, requiring lenses to study the connections among the biotic community, the land and animal cultures. This approach in turn integrates multiple ways of thinking about the environment into coevolving ethical technologies and resources. We unify these observations under ecogenomics, as an adaptable, cooperative and collective response to health. It is based on the interactions among human and nonhuman individuals in situ of biotic and abiotic factors—as well as their phenotypes and their genotypes—in the context of ecosystems. Our vision for the Ecological Genome Project is for it to develop in opportune ways if space is given for innovative collaborations. The fruits of interdisciplinarity will ripen, as knowledge cross‐fertilises expanded networks across more diverse places. The expanded vision is inclusive of sociocultural context, geography and environmental factors. It allows for the discovery of variants within and between, as well as the genetic ancestry of species, that are indicative of active and residual elements of ecosystems.

Our workshop started as a dialogue: connecting the familiar world (within HUGO) of ecogenetics to the uncharted one of ecogenomics. It was emphasised that ethics in the latter field had already been mapped to a large degree by ecologists, conservationists and veterinarians, as well as animal and environmental ethicists. It was acknowledged, too, that some views were missing from our workshop and indeed across the ELSI movement as a whole. Therefore, the workshop—guided by the momentum currently in OH—took just the first few steps in identifying connections between ecology and human genomics. It became clear to the authors that realising this vision will require further reflection on the implication for our particular disciplines, more debate, respectively, on the ethical issues that raises for each of us, identification of knowledge gaps, willingness to engage in unfamiliar discourses, and more community engagement. But it was also a place where experts, less siloed in their approach to sources of knowledge, undertook critical analysis *and* engaged with unfamiliar questions. We were left with the sense that it would be impossible to separate the human genome from the context of place and the lives with which we share this Planet.

## Data Availability

Data sharing is not applicable to this article as no data sets were generated or analysed during the current study.

## Endnotes

1 This is a modern reiteration, as One Medicine probably goes back to the early 1800's; Capps [15]; Easton and Alder [38].

2 That ‘encompasses life‐course environmental exposures (including lifestyle factors), from the prenatal period onwards’; see Wild [39]. Also see, Argentieri et al. [40].

3 These are called polygenic risk scores, or multifactorial likelihood estimates since they can include environmental factors as well as genetic ones. Jayasinghe et al. [41].

4 In their figure 3, mapping disciplinary expertise, the areas we focus on here are largely missing; Black et al. [58].

5 The US Agency for International Development's DEEP VZN project began in 2021 to sequence viruses that pose pandemic risk (it was a follow‐on from the PREDICT programme, 2009–2020). It was closed in 2023; Willan [60]. The Global Varome Project (2015) had similar ambitions and was closed down in 2020. Also see, Grange et al. [61].
